# Characterization of the complete chloroplast genome of *Sansevieria trifasciata var. Laurentii*

**DOI:** 10.1080/23802359.2020.1860717

**Published:** 2021-01-21

**Authors:** Weiyue Guo, Pan Li, Kang Lei, Lusha Ji

**Affiliations:** School of Pharmacy, Liaocheng University, Liaocheng City, Shandong Province, China

**Keywords:** *Sansevieria trifasciata*, Draceanaceae, phylogenetic, complete chloroplast genome

## Abstract

*Sansevieria trifasciata* var*. laurentii (S. trifasciata)* is a kind of popular in-door and out-door plant around world, it is not only known as the ornamental plant, but also as medical plant. It belongs to the *Draceanaceae* family, *Draceanaceae* includes more than 60 species distributed in tropical and subtropical dry climate regions. In this study, we sequenced the sample of *S. trifasciata* and determined its complete chloroplast genome. The length of CP genome is 155,179 bp, includes two invert repeats (IR) regions of 26,513 bp, a large single copy (LSC) region of 83,680 bp and a short single copy (SSC) region of 18,473 bp. There are 133 genes, which includes 87 protein coding genes, 8 rRNA and 38 tRNA, and 37.5% overall GC content. Each of *trn*K-UUU, *rps*16, *trn*G-UCC, *atp*F, *rpo*C1, *trn*L-UAA, *trn*V-UAC, *pet*B, *pet*D, *rpl*16, *rpl*2, *ndh*B, *trnI*-GAU, *trn*A-UGC and *ndh*A genes contains a intron, *clp*P and *ycf*3 contains 2 intron. The phylogenetic position shows that *S. trifasciata* has the closest relationship with *Rohdea Chinensis* (MH356725.1).

*Sansevieria trifasciata* var*. laurentii* (*S. trifasciata*) is a kind of popular in-door and out-door plant around world, however *S. trifasciata* is not only known as the ornamental plant, but also as medical plant. In traditional medical care, it was used for antitussive and an expectorant, and in South America it was sold as a crude drug in the market to treat victims of cough, snake bite, sprain, bruise, boil, abscess, respiratory inflammation and hair tonic(Stover 1983; Antunes et al. 2003) Furthermore, *S. trifasciata* is a kind of raw sauce of medicine (saponins) that can be used for tumor cell growth inhibitor and lowering blood cholesterol level. That medicine can also be used as spermicide (contraception), anti-inflammation, cytotoxic and antimicrobial agents (Dey 2014; Odeh and Amom 2014). *Sansevieria trifasciata* belongs to the *Draceanaceae* family, which includes more than 60 species distributed in tropical and subtropical dry climate regions (Lu and Morden 2014). Base the situation that no complete chloroplast genome of the species in *Sansevieria* has been published, so in this study we sequenced the sample of *S. trifasciata* and determined its complete chloroplast genome.

The sample of *S. trifasciata* was collected from South China Botanical Garden, Tianhe District, Guangzhou, Guangdong Province (N113°22′34ʺ, E23°11′32ʺ). We used the fresh leaves to extract chloroplast DNA based CTAB method (Doyle and Doyle 1987) and constructed the libraries with an average length of 350 bp using the NexteraXT DNA Library Preparation Kit (Illumina, San Diego, CA). Then the libraries were sequenced on Illumina Novaseq 6000 platform, over 2 Gb clean data was assembled by SPAdes v.3.11.0 software (Bankevich et al. 2012) and annotated by GeSeq (Tillich et al. 2017). The complete sequence and annotation results were submitted to GenBank, under the accession number (MT922036) and the sample was stored at Laboratory of Molecular Biology, Liaocheng University, Liaocheng (Voucher specimen: ST20200701LP).

The complete chloroplast genome of *S. trifasciata* is 155,179 bp in length, and contains a large single copy (LSC) with 83,680 bp in length, a small single copy (SSC) with 18,473 bp in length and two inverted repeat (IR) regions of 26,513 bp each. There were 133 genes, which includes 87 protein coding genes, 8 rRNA and 38 tRNA, and 37.5% overall GC content. Each of t*rn*K-UUU*, rps*16*, trn*G-UCC*, atp*F*, rpo*C1*, trn*L-UAA*, trn*V-UAC*, pet*B*, pet*D*, rpl*16*, rpl*2*, ndh*B*, trn*I-GAU*, trn*A-UGC and *ndh*A genes contains a intron, *clp*P and *ycf*3 contains 2 introns.

To confirm the phylogenetic position and understand the relationship of *S. trifasciata*. The complete chloroplast genome of 15 species were collected and aligned with *S. trifasciata* by MAFFT7.037 (Katoh and Standley 2013). Subsequently, the phylogenetic tree was constructed by IQTREE v1.6 (Nguyen et al. 2015; Hoang et al. 2018) with 1000 bootstraps replicates using Best-fit model. By using *Hemerocallis fulva* (LC554221.1) as out group we got the final ML tree, then Figure 1 showed that *S. trifasciata* had the closest relationship with *Rohdea chinensis* (MH356725.1).

**Figure 1.**
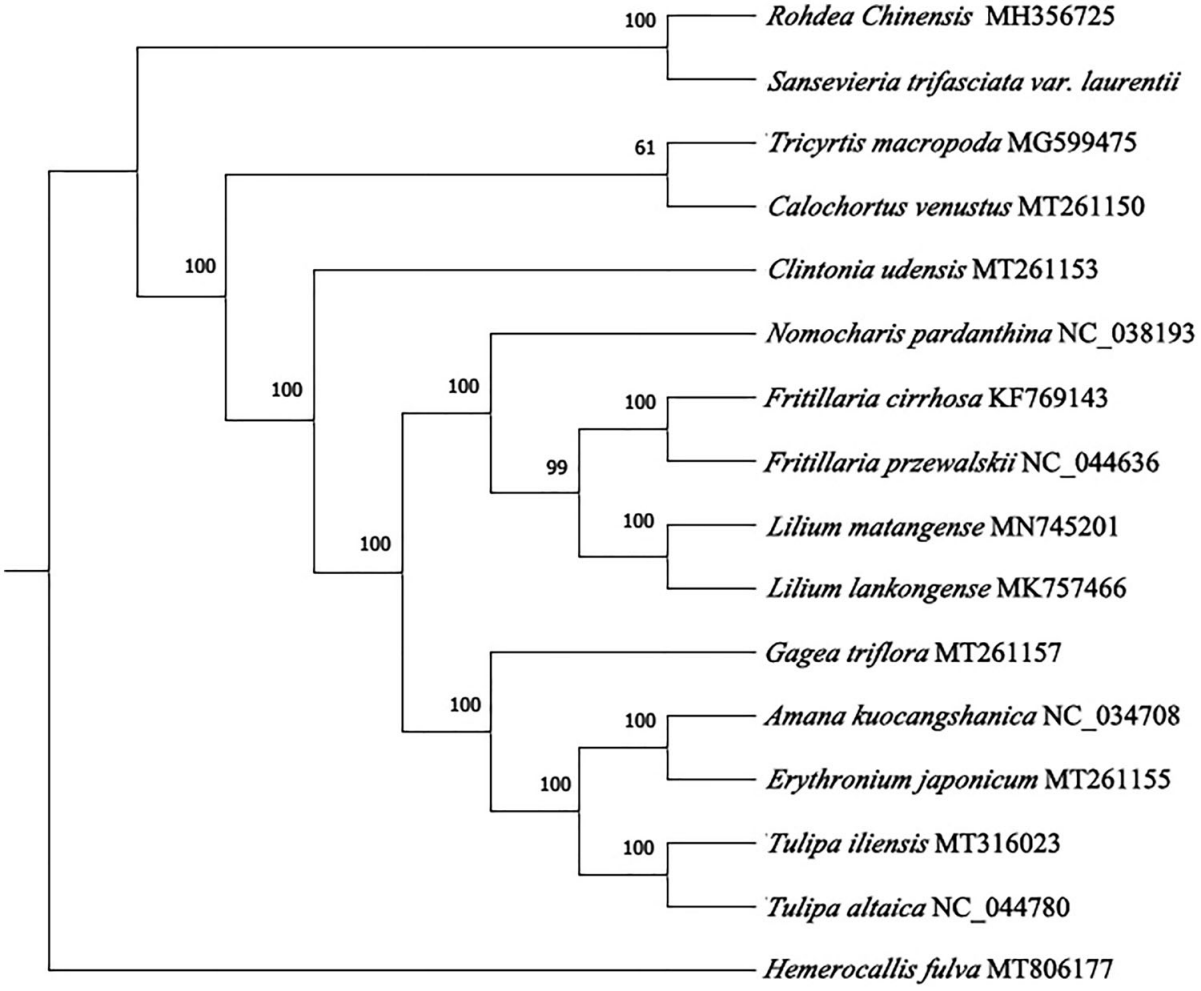
Maximum-likelihood phylogenetic tree for *S. trifasciata* based on 16 complete chloroplast genomes. The Genbank accession numbers are on the diagram.

## Data Availability

The sequencing data that support the finding of this study are openly available in NCBI Sequence Read Archive(SRA) with accession number: SRR12980945. The assembled complete chloroplast genome sequence of *Sansevieria trifasciata* var*. laurentii* has been submitted to GenBank under the accession number: MT922036 (https://www.ncbi.nlm.nih.gov/nuccore/MT922036.1).
